# Analyzing the characteristics of respiratory microbiota after the placement of an airway stent for malignant central airway obstruction

**DOI:** 10.1128/spectrum.03472-23

**Published:** 2024-05-15

**Authors:** Yue Wang, Yunzhi Zhou, Yan Huang, Xiaoli Li, Jieli Zhang, Yongping Gao, Fang Qin, Huaixiu Fu, Shufang Wang, Anan Niu, Ruinan Guo

**Affiliations:** 1 Graduate School of North China University of Technology, Tangshan, China; 2 Department of Respiratory and Critical Care Medicine, Emergency General Hospital, Beijing, China; 3 Department of Respiratory and Critical Care Medicine, North China University of Science and Technology Affiliated Hospital, Tangshan, China; University of Arkansas Fayetteville, Fayetteville, Arkansas, USA

**Keywords:** lung microbiota, airway stent, malignant central airway obstruction, metagenomic next-generation sequencing

## Abstract

Malignant central airway stenosis is treated with airway stent placement, but post-placement microbial characteristics remain unclear. We studied microbial features in 60 patients post-stent placement, focusing on changes during granulation tissue proliferation. Samples were collected before stent (*N* = 29), after stent on day 3 (*N* = 20), and after granulation tissue formation (AS-GTF, *N* = 43). Metagenomic sequencing showed significant respiratory tract microbiota changes with granulation tissue. The microbiota composition, dominated by *Actinobacteria*, *Firmicutes*, and *Proteobacteria*, was similar among the groups. At the species level, the AS-GTF group exhibited significant differences, with *Peptostreptococcus stomatis* and *Achromobacter xylosoxidans* enriched. Analysis based on tracheoesophageal fistula presence identified *Tannerella forsythia* and *Stenotrophomonas maltophilia* as the main differential species, enriched in the fistula subgroup. Viral and fungal detection showed *Human gammaherpesvirus 4* and *Candida albicans* as the main species, respectively. These findings highlight microbiota changes after stent placement, potentially associated with granulation tissue proliferation, informing stent placement therapy and anti-infective treatment optimization.

**IMPORTANCE:**

Malignant central airway stenosis is a life-threatening condition that can be effectively treated with airway stent placement. However, despite its clinical importance, the microbial characteristics of the respiratory tract following stent insertion remain poorly understood. This study addresses this gap by investigating the microbial features in patients with malignant central airway stenosis after stent placement, with a specific focus on microbial changes during granulation tissue proliferation. The findings reveal significant alterations in the diversity and structure of the respiratory tract microbiota following the placement of malignant central airway stents. Notably, certain bacterial species, including *Peptostreptococcus stomatis* and *Achromobacter xylosoxidans*, exhibit distinct patterns in the after-stent granulation tissue formation group. Additionally, the presence of tracheoesophageal fistula further influences the microbial composition. These insights provide valuable references for optimizing stent placement therapy and enhancing clinical anti-infective strategies.

## INTRODUCTION

Malignant central airway stenosis is a common respiratory disease, mainly caused by malignant tumors invading or compressing the airway, resulting in symptoms such as dyspnea and even threatening the patient’s life (1). Airway stent placement is an effective treatment method, which can relieve airway stenosis and dyspnea, but it may also change the characteristics of the airway microbiota, increasing the risk of respiratory infections and other complications (2).

Numerous studies have confirmed that the microbial composition varies in different disease states such as bronchial asthma (3), chronic obstructive pulmonary disease (COPD) (4), and lung cancer (5). Different states of the same disease, such as asthma exacerbation (6), different severity levels of COPD (7), and COPD exacerbation (8), also precipitate changes in the respiratory microbiota. As a foreign body, the airway stent changes the characteristics of the respiratory microbiota after placement (9) and may cause the overgrowth of potential pathogens, leading to complications such as respiratory infections (10). However, a limited number of studies have investigated the alterations in respiratory microbiota after stent placement, leaving some unresolved issues.

This study aims to explore the changes in respiratory microbiota characteristics after stent placement in patients with malignant central airway stenosis by metagenomics next generation sequencing (mNGS). It provides new ideas and references for the optimization and improvement of stent placement treatment and clinical anti-infection treatment and reveals the dynamic changes of respiratory microbiota after stent placement, especially the microbiota characteristics of granulation tissue formation. We hope to discover new therapeutic targets and intervention strategies to reduce the complications after stent placement.

## RESULTS

### Patients’ characteristics

After sequencing, a total of 92 bronchoalveolar lavage fluid (BALF) samples from 60 patients were included for subsequent analysis. We categorized them into three groups according to the collection time: before-stent group (BS, *N* = 29), after-stent day 3 group (AS-D3, *N* = 20), and after-stent granulation tissue formation group (AS-GTF, *N* = 43). The clinical characteristics for all three groups showed no significant differences (Table 1). In AS-GTF, 29 of the samples had pneumonia. The conventional culture methods yielded a total of 12 positive cases, comprising one instance of *Pseudomonas putida*, one instance of *Proteus vulgaris*, one instance of *Morganella morganii*, two instances of *Acinetobacter baumannii*, one instance of *Klebsiella pneumoniae*, and six instances of *Pseudomonas aeruginosa*.

**TABLE 1:** Baseline characteristics of study subjects^*a*^

	BS (*N* = 29)	AS-D3 (*N* = 20)	AS-GTF (*N* = 43)	Statistic value	*P* value
Patients/samples	29/29	20/20	35/43		
Sex (female)	5 (17.2)	4 (20)	10 (23.3)	*χ*^2^ = 0.389	0.849
Age (years)	65.2 ± 10.0	58.5 ± 9.6	60.2 ± 12.2	*F* = 2.679	0.074
Primary disease				χ^2^ =2.152	0.720
Pulmonary malignancies	11 (37.9)	8 (40.0)	20 (46.5)		
Squamous cell carcinoma	7	6	9		
Adenocarcinoma	1	1	5		
Small cell carcinoma	3	0	1		
Carcinoids	0	0	3		
Adenoid cystic carcinoma	0	1	2		
Esophageal cancer	15 (51.7)	8 (40.0)	19 (44.2)		
Other malignancies	3 (10.3)	4 (20.0)	4 (9.3)		
Laryngeal cancer	0	1	0		
Lymphoma	0	1	0		
Malignant tumor of mediastinum	3	2	1		
Osteosarcoma	0	0	1		
Thyroid cancer	0	0	2		
Combined with TEF	6 (20.7)	3 (15.0)	13 (30.2)	χ^2^ = 1.983	0.391
Tumor stage				χ^2^ = 1.494	0.883
Stage I + II	1 (3.4)	1 (5.0)	3 (7.0)		
Stage III + IV	23 (79.3)	15 (75.0)	35 (81.4)		
Stage unknown	5 (17.2)	4 (20.0)	5 (11.6)		
Stent type				χ^2^ = 1.660	0.837
Silicone stent	3 (10.3)	4 (20.0)	5 (11.6)		
Y type	1	1	1		
Fully covered metallic stent	22 (75.9)	14 (70.0)	34 (79.1)		
Y type	21	12	26		
Ultraflex stent	4 (13.8)	2 (10.0)	4 (9.3)		
Diabetes	6 (20.7)	4 (20.0)	8 (18.6)	χ^2^ = 0.051	1.000
Immunological drug use within 1 year	10 (34.5)	8 (40.0)	15 (34.9)	χ^2^ = 0.191	0.921
Pneumonia	17 (58.6)	13 (65.0)	29 (67.4)	χ^2^ = 0.594	0.721
Antibiotic	12 (41.4)	13 (65.0)	24 (55.8)	χ^2^ = 2.864	0.239
Third-generation cephalosporins	10	10	19		
With fluconazole	2	1	0		
Others	2	3	5		
Moxifloxacin	0	0	2		
Carbapenem	2	3	2		
Piperacillin/tazobactam	0	0	1		

^*a*^ Data are the mean ± SD or *n* (%) as appropriate.

### Analyzing bacterial microbiota in different groups

**Fig 1:**
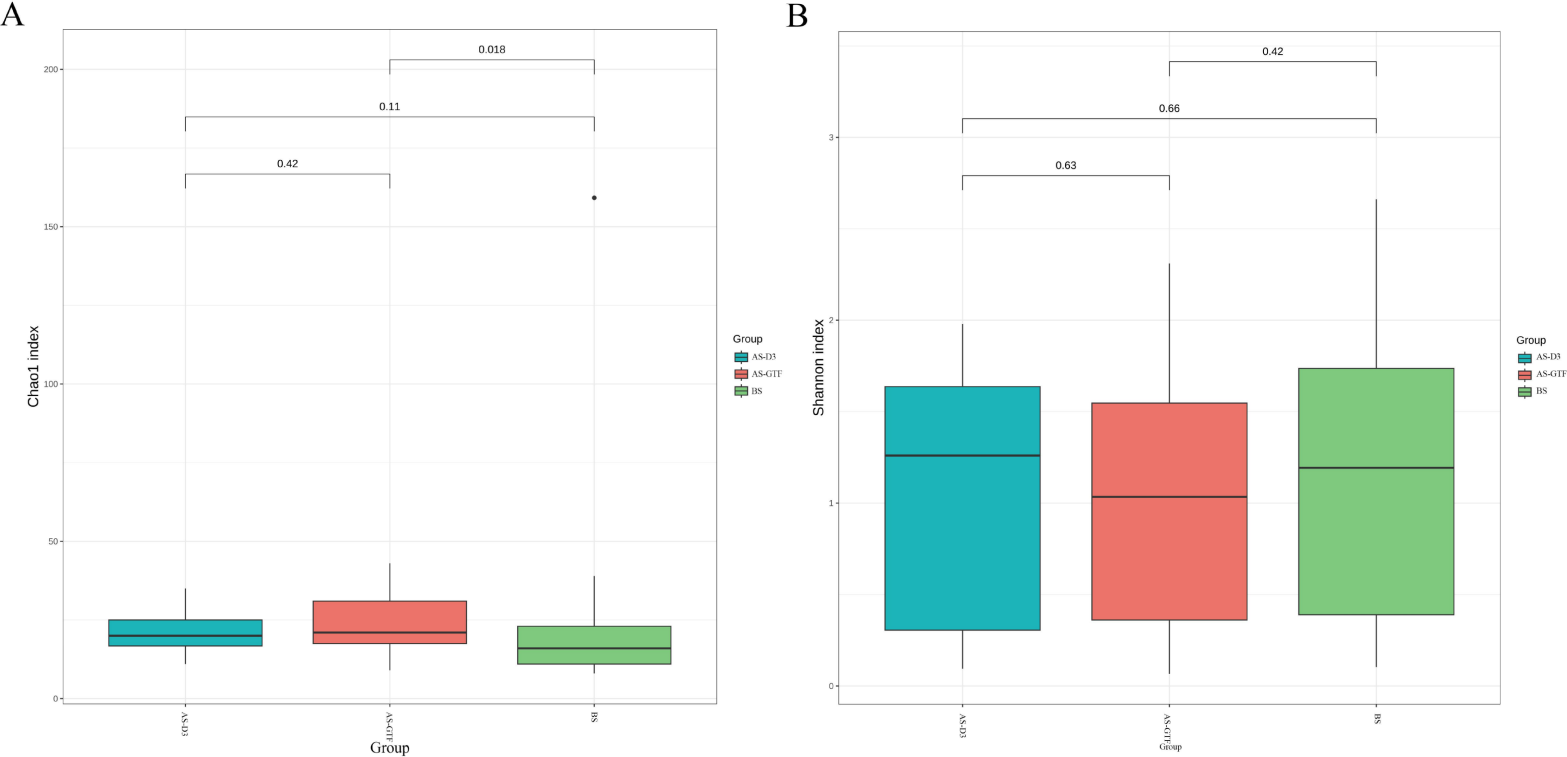
Taxonomic alpha diversity of bacterial microbiota within samples in different groups. Comparison of Chao1 index (A) and Shannon index (B) among the BS, AS-D3, and AS-GTF groups. The Chao1 index revealed a significant difference between the BS group and the AS-GTF group.

**Fig 2:**
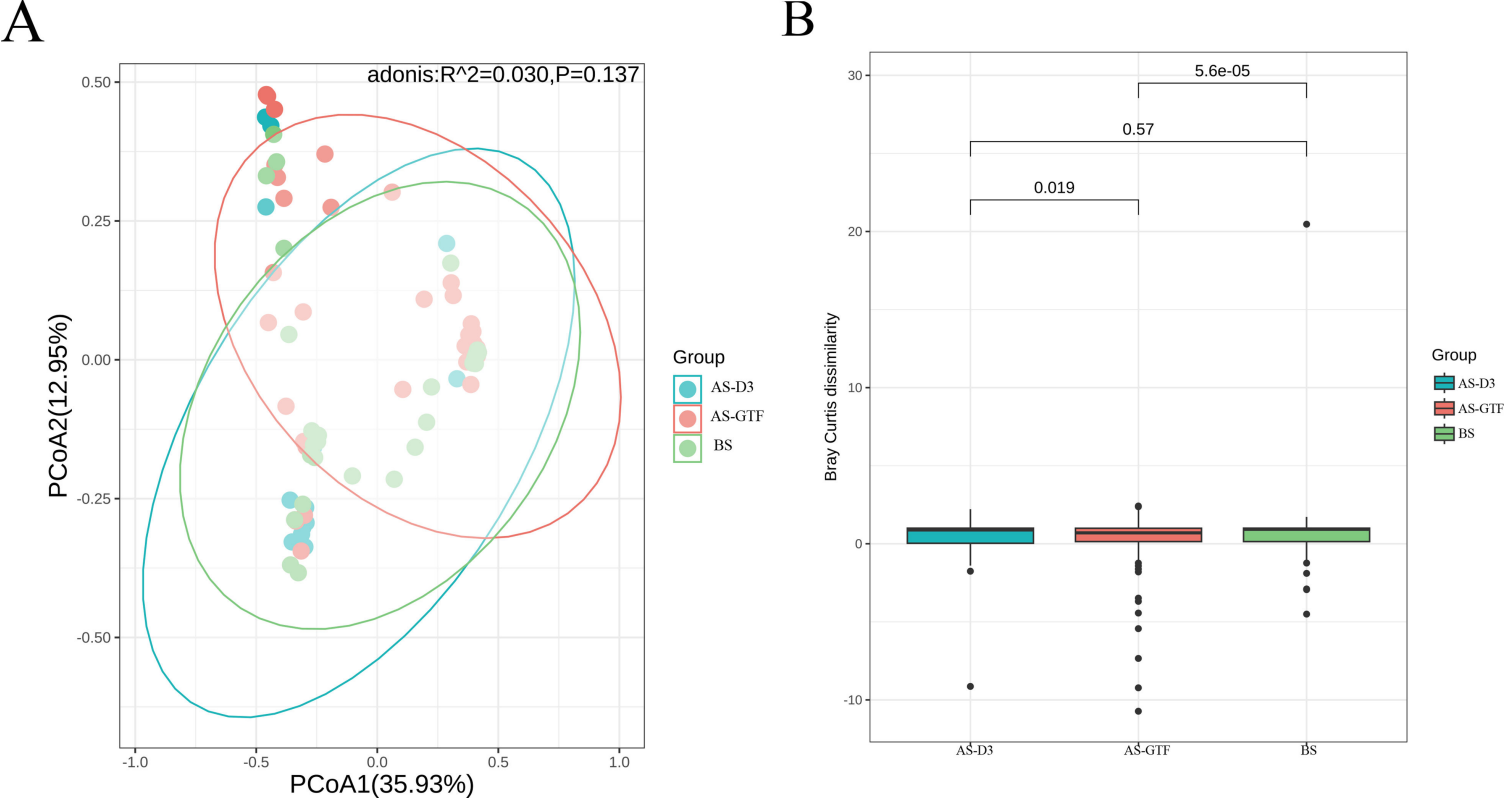
Comparison of bacterial community structure. PCoA (A) and Bray-Curtis dissimilarity (B) among the BS, AS-D3, and AS-GTF groups. Bray-Curtis dissimilarity shows that the AS-GTF group is significantly different from the other two groups.

We analyzed the respiratory microbiota in three groups. The goal was to explore how stent placement impacts respiratory microbiota. The Chao1 index revealed a significant difference between the BS group and the AS-GTF group (*P* = 0.018), whereas the Shannon index showed no significant variation among the three groups (Fig. 1). The analysis of the bacterial community structure revealed a significant difference in AS-GTF compared to the other two groups, as demonstrated by the Bray-Curtis distance analyses. Nonetheless, the principal coordinates analysis (PCoA) did not indicate a significant separation (Fig. 2). This suggests that the diversity and structure of respiratory microbiota may undergo significant changes when granulation tissue formation occurs after airway stent placement.

**Fig 3:**
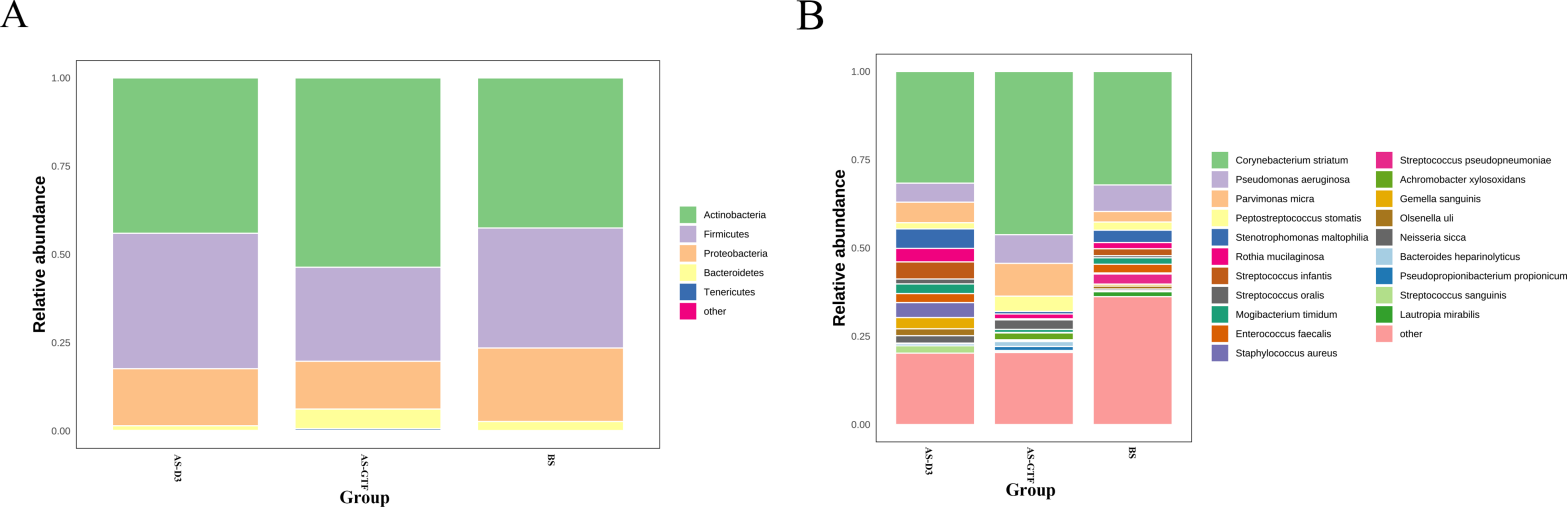
Bacterial microbiota composition at the phylum (A) and species (B) level in the BS, AS-D3, and AS-GTF groups.

**Fig 4:**
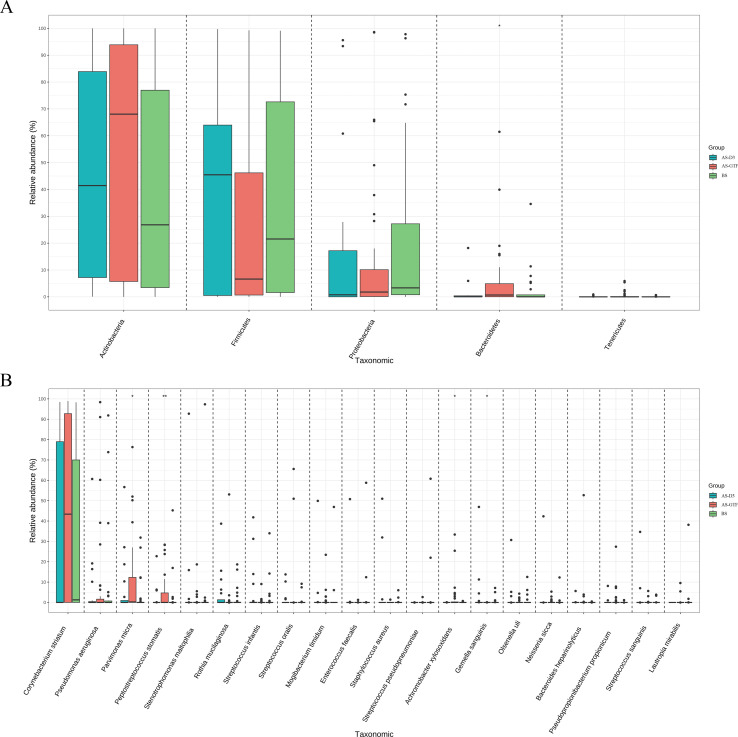
Comparison of relative abundance at the phylum (A) and species (B) level in the BS, AS-D3, and AS-GTF groups. *P < 0.05 and **P <0.01.

Analyzing the microbiota composition revealed similarities and differences across the three groups. The BS, AS-D3, and AS-GTF groups exhibited comparable bacterial community composition at the phylum level, with *Actinobacteria* being the predominant phylum in all groups (42.51%, 44.01%, and 53.64%, respectively). Additionally, *Firmicutes* accounted for a substantial proportion in each group (33.99%, 38.35%, and 26.62%, respectively), followed by *Proteobacteria* (20.81%, 16.16%, and 13.53%, respectively), and *Bacteroidetes* (2.57%, 1.34%, and 5.55%, respectively) (Fig. 3A). However, at the species level, there were slight differences in the main species composition. The BS group was primarily composed of *Corynebacterium striatum, Pseudomonas aeruginosa*, and *Stenotrophomonas maltophilia*. On the other hand, the AS-D3 group mainly included *Corynebacterium striatum, Parvimonas micra,* and *Stenotrophomonas maltophilia*. Similarly, the AS-GTF group mostly consisted of *Corynebacterium striatum, Parvimonas micra, and Pseudomonas aeruginosa* (Fig. 3B). Despite these similarities, there were significant differences at the phylum, class, order, family, genus, and species levels. Specifically, significant differences were observed in the phylum of *Bacteroidetes* and in the species of *Peptostreptococcus stomatis, Achromobacter xylosoxidans, Parvimonas micra, and Gemella sanguinis* (Fig. 4). In the AS-GTF group, *Peptostreptococcus stomatis*, *Achromobacter xylosoxidans*, and *Parvimonas micra* showed significant differences compared to the BS group and the AS-3D group.

**Fig 5:**
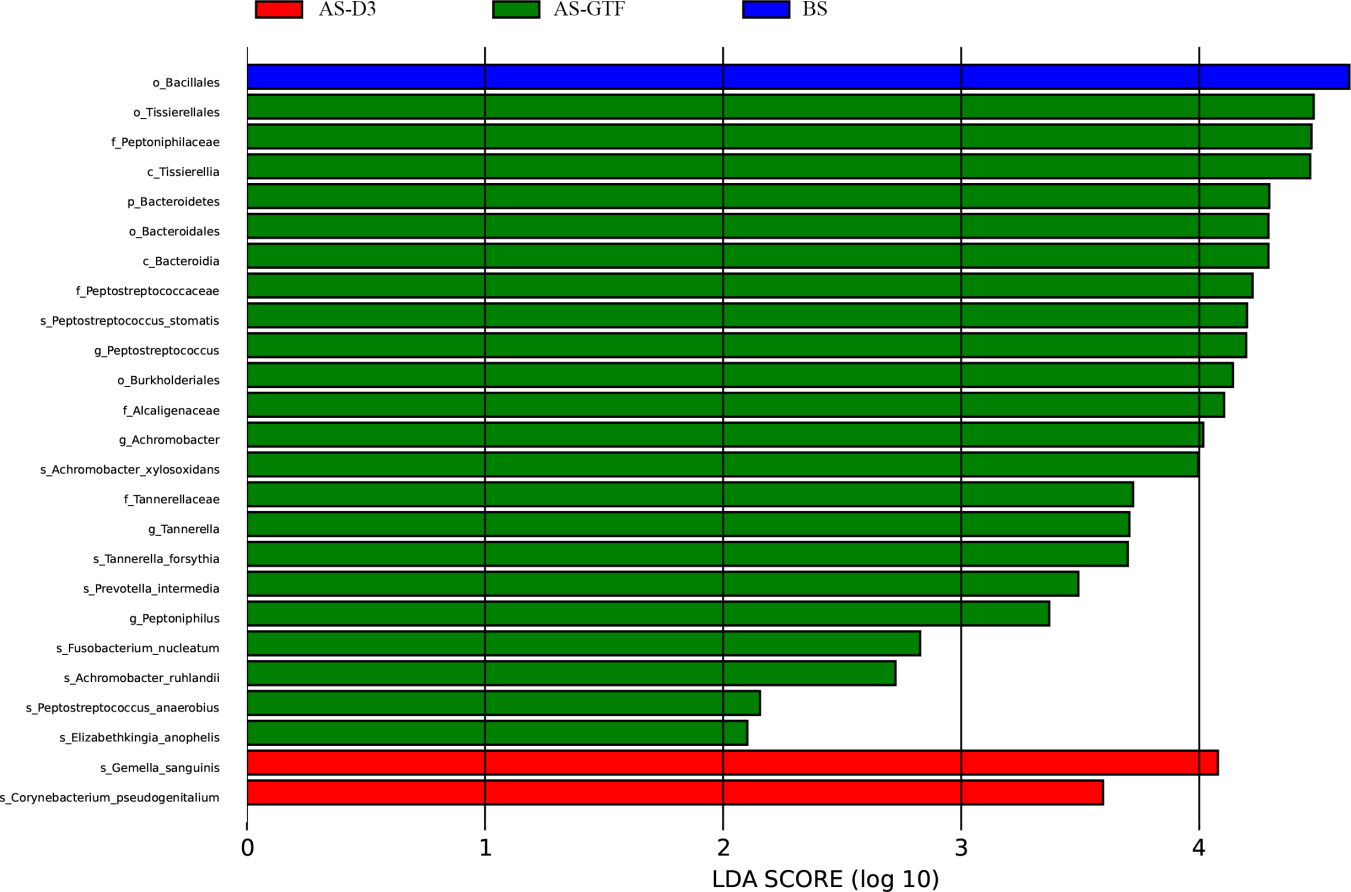
Differential taxa among the BS, AS-D3, and AS-GTF groups by LEfSe analysis based on the LDA score.

We employed linear discriminant analysis effect size (LEfSe) to identify species with biomarkers. The LEfSe analysis indicated that, with a linear discriminant analysis (LDA; log10) threshold of 2.0, 22 differential taxa were identified in the AS-GTF group. These taxa include *Peptostreptococcus stomatis, Achromobacter xylosoxidans, Tannerella forsythia, Prevotella intermedia, Fusobacterium nucleatum, Achromobacter ruhlandi, Peptostreptococcus anaerobius,* and *Elizabethkingia anophelis* at the species level (Fig. 5).

### Analyzing fungi and viruses in different groups

In the BS, AS-D3, and AS-GTF groups, some samples exhibited the presence of fungi. Of these, 14 cases were found in the BS group, 7 in the AS-D3 group, and 19 in the AS-GTF group. We conducted a diversity analysis of the microbiota among the three groups, which revealed no significant diversity difference. In terms of phylum, the composition of the microbiota primarily consisted of *Ascomycota, Basidiomycota,* and *Mucoromycota*. At the species level, the fungi predominantly found were *Candida albicans, Aspergillus fumigatus, Candida tropicalis,* and *Candida parapsilosis,* with *Candida albicans* being the most dominant (Fig. S1). However, the analysis did not yield any statistically significant differences in species composition among the three groups.

**Fig 6:**
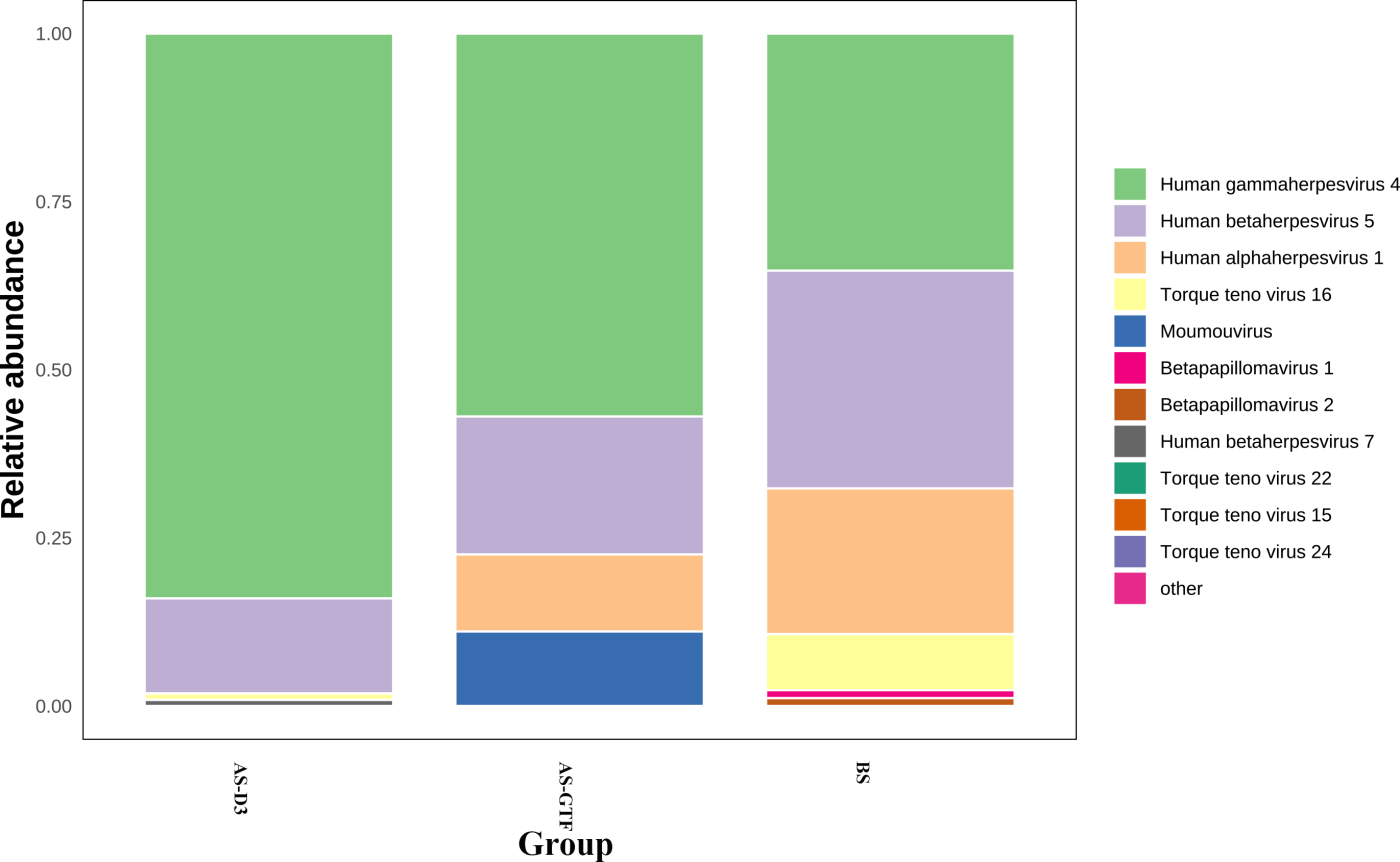
Virus microbiota composition at the species level in the BS, AS-D3, and AS-GTF groups.

We also undertook an analysis of the viral presence within the samples from the three groups. The results indicated that 12 cases of viruses were detected in the BS group, 7 in the AS-D3 group, and 9 in the AS-GTF group. The primary viruses detected were *Human gammaherpesvirus 4, Human betaherpesvirus 5,* and *Human alphaherpesvirus 1* (Fig. 6). These viruses are part of the *human herpesvirus* family, with *Human gammaherpesvirus 4* also known as Epstein-Barr virus (EBV)*, Human betaherpesvirus 5* as cytomegalovirus*,* and *Human alphaherpesvirus 1* as herpes simplex virus 1 (HSV-1). The occurrence of these viruses in the respiratory microbiota following stent implantation could be linked to the patient’s immune status and pathological process. However, we are yet to ascertain the specific function and effect of these viruses on the patient’s health post-stent implantation.

### Analyzing respiratory microbiota in AS-GTF based on the presence or absence of tracheoesophageal fistula

**Fig 7:**
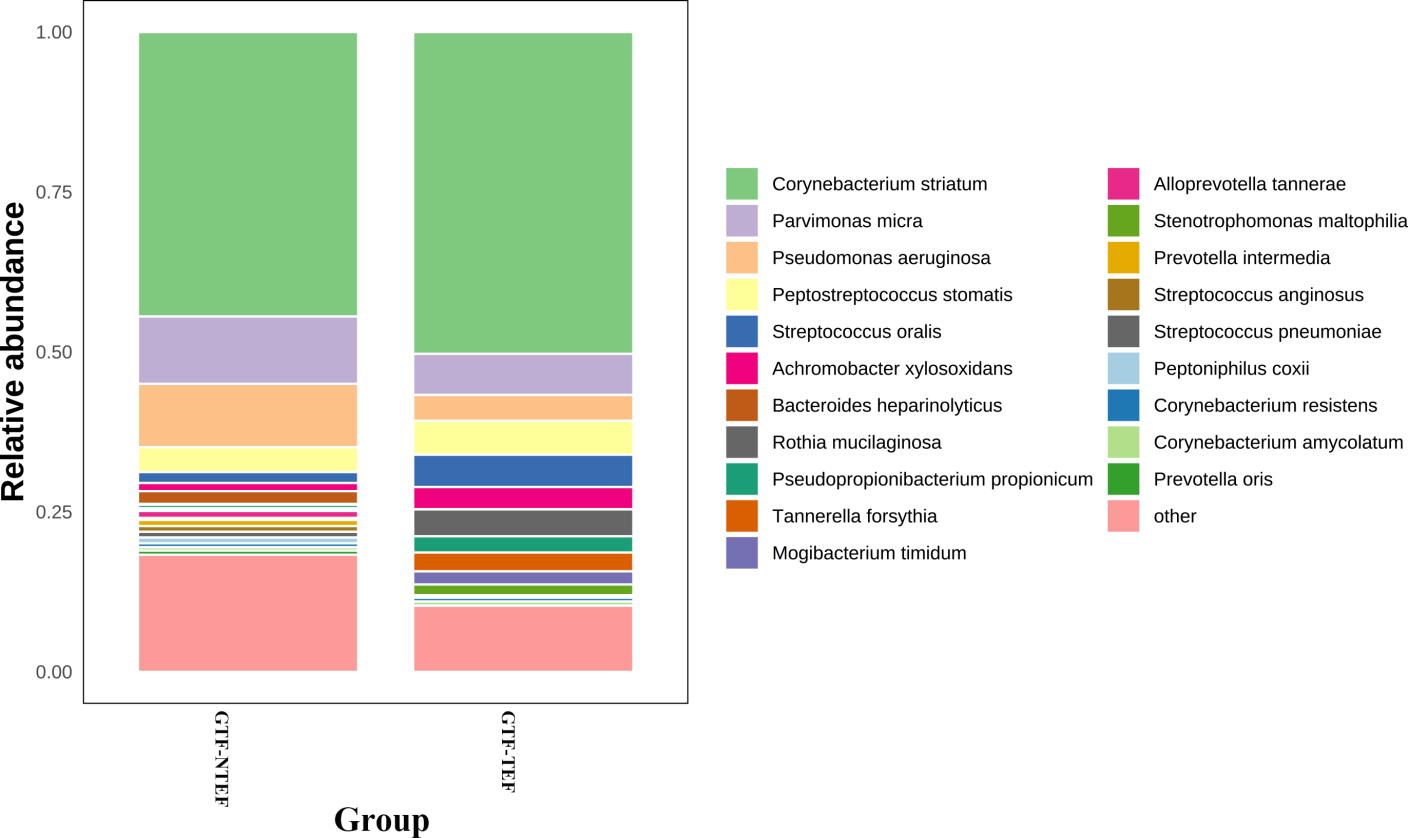
Relative abundance of bacteria at the species level in the TEF and NTEF subgroups.

**Fig 8:**
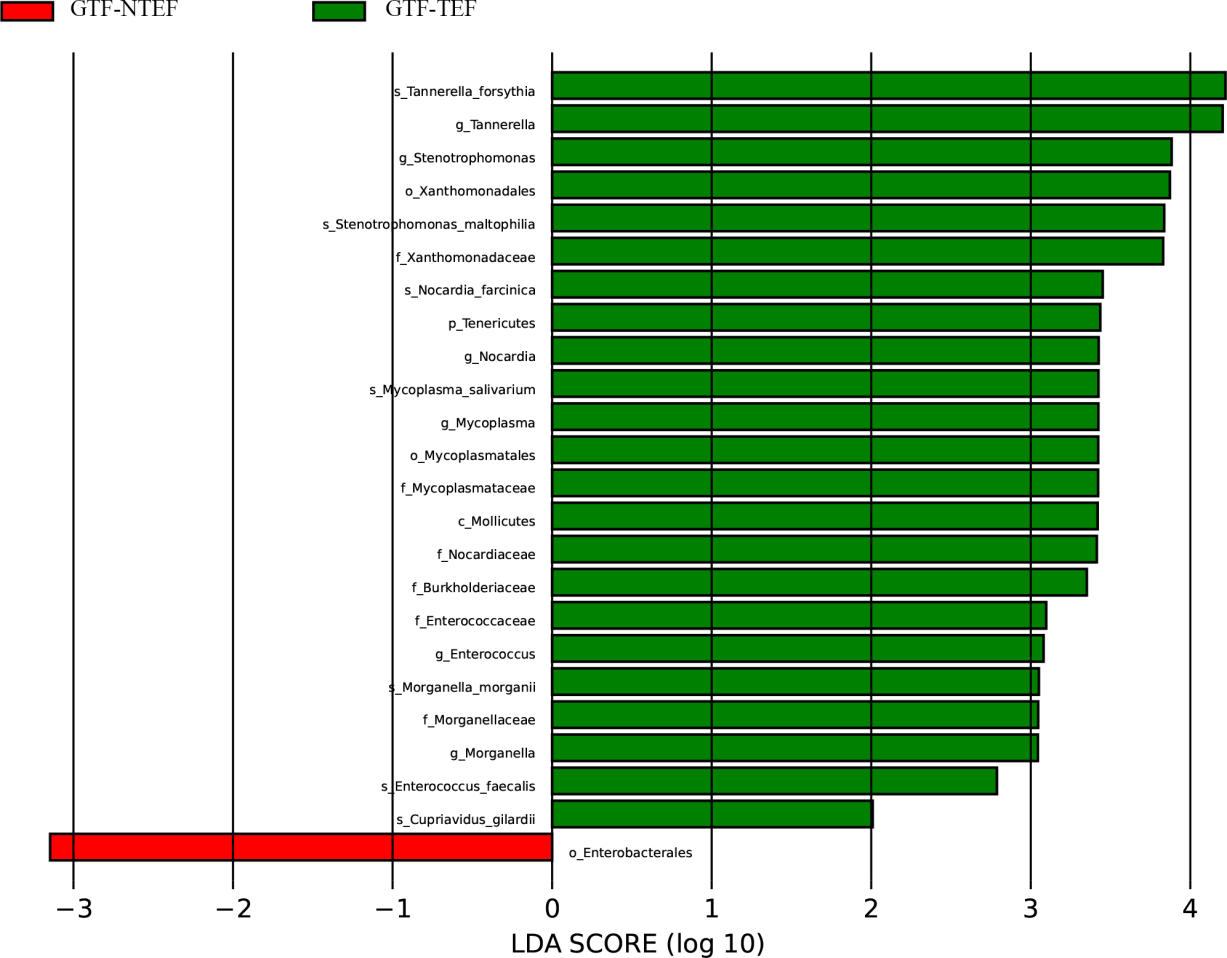
Differential taxa in the TEF and NTEF subgroups by LEfSe analysis based on the LDA score.

We categorized the AS-GTF group into two subgroups based on the presence or absence of tracheoesophageal fistula (TEF). The first subgroup included 13 cases with TEF, while the second subgroup consisted of 30 cases without TEF (NTEF). An analysis of the bacterial communities in these subgroups revealed no significant differences in the ACE, Chao1, Shannon and Simpson indices, as well as NMDS, PCA, and PCoA indicators. This finding indicates that there is a comparable microbial diversity and structure in the respiratory tract following stent implantation among both subgroups. The two groups exhibited a shared microbial composition, primarily consisting of *Corynebacterium striatum*, *Parvimonas micra*, *Pseudomonas aeruginosa*, and *Peptostreptococcus stomatis* (Fig. 7). However, there were some disparities found in the differential microbiota analysis. Specifically, significant differences were observed in the phylum of *Tenericutes* and in the species of *Tannerella forsythia* and *Stenotrophomonas maltophilia*. Moreover, a LEfSe analysis revealed that the subgroup TEF had more biomarkers in the bacterial community (Fig. 8).

We conducted additional fungal analysis on the two subgroups, revealing six instances in the TEF group and 13 in the NTEF group. The primary fungi at the phylum level were *Ascomycota*, succeeded by *Basidiomycota* in the NTEF group and *Mucoromycota* in the TEF group. As for the species level, *Candida albicans* was the predominant fungal species, detected in three cases in the TEF subgroup and two cases in the NTEF subgroup (Fig. S2). Alongside this, our viral analysis identified eight cases of viruses in the NTEF group and only one in the TEF group, with *Human gammaherpesvirus 4* being the most frequently detected virus.

## DISCUSSION

This study used mNGS technology to conduct a cross-sectional study on the respiratory microbiota characteristics before and after airway stent placement and included 92 BALF samples. We found that the lower respiratory microbiota changed when granulation tissue formation occurred after airway stent placement compared to before stent placement. At the species level, the three groups had similar but slightly different compositions of species. We screened out bacteria related to granulation tissue formation after stent placement by LEfSe analysis and found that there were more biomarkers in the AS-GTF group with concomitant TEF.

Previous beliefs held that the lower respiratory tract was sterile unless infected. However, advancements in detection technology have revealed the unique characteristics of lower respiratory tract microbiota. In healthy individuals, the microbiota is primarily composed of *Firmicutes*, *Proteobacteria*, *Bacteroidetes*, *Actinobacteria*, and *Fusobacteria* at the phylum level (11, 12). However, this composition changes significantly under different disease states (12–15). The composition of respiratory microbiota is chiefly influenced by microbial migration, microbial elimination, and the relative growth rate of its members. In healthy patients, microbial migration and elimination are the main factors, while for patients with advanced lung cancer, the characteristics of lower respiratory tract microbiota are mainly determined by the main regional growth conditions (16). At present, there are few studies on the characteristics of the respiratory microbiome after malignant airway stenosis and implantation of airway stents. This study found that patients with malignant central airway stenosis were mainly composed of bacteria from four phyla: *Actinobacteria*, *Firmicutes*, *Proteobacteria*, and *Bacteroidetes*. This is in line with previous research, though the order is slightly different (*Firmicutes*, *Proteobacteria*, *Actinobacteria*, and *Bacteroidetes*) (12), which may be due to the patient’s airway stenosis status and treatment method.

Airway stent implantation is a crucial treatment for malignant central airway stenosis (17). Simple bronchoscopy examination without airway stent implantation is beneficial for clearing airway-colonizing bacteria and preventing infection (9), and the change of microbiota after stent implantation is mainly caused by the stent itself. Our findings suggest that the relative abundance and composition of microbiota may change at different stages post-stent implantation. The microbiota composition of the BS, AS-D3, and AS-GTF groups was similar but slightly different. This may be related to two factors: first, the implantation of an airway stent can lead to airway injury, creating bacterial binding sites in the bronchial tree’s basement membrane, which can stimulate mucus secretion (18). Second, the difficulty in discharging mucus after stent implantation can create potential sites for bacterial attachment, and the irregular surface of the stent can also facilitate bacterial colonization (18).

Granulation tissue formation is a common complication after airway stent implantation (19), which can affect the efficacy of the treatment and lead to severe cases of respiratory failure and death. Microbial colonization or infection plays a pivotal role in the process of granulation tissue formation following the implantation of an airway stent, and it is noteworthy that the formation of granulation tissue can be substantially mitigated through effective anti-infective treatment (20). Previous studies have demonstrated the feasibility of microbiota detection in the tracheostomy tube through metagenomic sequencing. Moreover, these studies have revealed significant distinctions in microbiota characteristics between patients with and without the formation of granulation tissue (21). In this study, the specimens with granulation tissue formation were almost all more than 1 week after stent implantation, and the respiratory microbiota characteristics changed significantly at this time. In the AS-GTF group, the majority of stents were fully covered metallic stents, with *Bacillus* being the predominant genus. Interestingly, the proportion of *Staphylococcus* was relatively low, which contrasts with prior reports based on 16sRNA sequencing, where fully covered metallic stents were primarily associated with *Staphylococcus* (22). This discrepancy may be attributed to the methodology employed in the original study, which relied on 16sRNA sequencing, whereas our current study utilized mNGS detection. Furthermore, it is worth noting that our study encompassed various types of stents, including Ultraflex stents and silicone stents, potentially contributing to variations in the microbiota composition. When granulation tissue formation occurs subsequent to stent implantation, there is a notable increase in the abundance of airway microbiota, accompanied by the emergence of certain statistically significant differential species. The majority of these differential species primarily consist of oral-colonizing bacteria and opportunistic pathogens. Previous research has substantiated the presence of oral bacteria within the microbial community of lung tissue. Importantly, this phenomenon is not attributed to contamination resulting from bronchoscopy insertion but rather occurs as a result of inhalation (7). Therefore, we propose that the presence of these oral-colonizing bacteria and opportunistic pathogens holds considerable importance; nevertheless, additional investigation is essential to confirm their significance. Previously, it was hypothesized that granulation tissue formation following stent implantation might be linked to infections involving *Staphylococcus aureus* and *Pseudomonas aeruginosa* (23–25). In our findings, 67.44% of the group with granulation tissue formation experienced respiratory tract infections. Surprisingly, fewer than half of the lavage fluid samples yielded positive results using conventional culture methods, with *Pseudomonas aeruginosa* being the predominant pathogen. These findings align with prior research results. However, we screened for bacterial communities associated with granulation after stent placement by using mNGS and LEfSe analysis on the lavage fluid. These communities were related to oral microbiota. It has been reported that the increase in *Prevotella* abundance is associated with pulmonary Th17 immune response (26), suggesting that these microbial communities may interact with the pulmonary immune system. The dysbiosis of lower respiratory tract microbiota characterized by oral-related microbiota enrichment enhances the host inflammatory response (27). The inflammatory response triggers local edema, leading to an enlargement of the originally well-fitted stent. This escalates mechanical exposure to the airway wall, including increased friction and contact pressure, ultimately resulting in the formation of granulation tissue. Granulation tissue subsequently results in airway obstruction, thereby exacerbating the blockage of mucus discharge, initiating modifications in the local microbiota (28), and establishing a detrimental cycle. The traditional culture method relies on the culture medium to determine the cultivable microorganisms, whereas mNGS technology has demonstrated its superiority over traditional culture in pathogen detection (29, 30). The comprehensive detection results obtained through mNGS technology enable a better understanding of the microbiota’s characteristics. Hence, unlike previous results, it appears that certain oral-colonizing bacteria and opportunistic pathogens, which are often underestimated, may indeed play a crucial role in the development of granulation tissue following stent implantation. Therefore, our findings provide a new perspective and a new framework for studying granulation tissue formation, which can help elucidate the underlying mechanisms and interactions of these factors.

We conducted a more in-depth analysis of fungi and viruses and discovered that 43.49% of the specimens contained fungi, while 9.78% of the specimens contained viruses, neither of which were detected through traditional bacteriological culture methods. Interestingly, there was no statistically significant difference in the presence of fungi among the three groups. The most commonly identified fungus is *Candida albicans*, aligning with previous research findings (31, 32). The primary viruses detected included *Human gammaherpesvirus 4*, *Human betaherpesvirus 5*, and *Human alphaherpesvirus 1. Human gammaherpesvirus 4*, commonly known as EBV, was initially identified through electron microscopy in Burkitt lymphoma cells (33). It primarily spreads through saliva. While its carcinogenic potential is relatively low, it is widespread and contributes to approximately 1.5% of global cancer cases (34), including oral squamous cell carcinoma (35). In our specimens, we detected six cases of EBV infection, which merits our attention. The association between EBV and lung malignancies remains unclear due to the limited sample size, highlighting the need for further research. Additionally, HSV has been reported as a potential cause of tracheoesophageal fistula (36).

When we conducted microbiota analysis on the subgroups with TEF and NTEF, we observed no significant differences in diversity indices between the two subgroups. However, variations in microbiota composition were evident, particularly when analyzed at the family, genus, and species levels. Notably, the analysis revealed distinct species differences among the subgroups. The LEfSe analysis results showed that the subgroup with TEF had more biomarkers. These microbiota may play a significant role in disease development and progression. *Candida albicans* exhibited a higher detection rate in the TEF subgroup. This can be attributed to the entry of digestive fluids and food into the airway, which elevates the risk of lung infections, weakens pulmonary defense mechanisms, and creates an opportunity for *Candida albicans* invasion. Furthermore, patients experiencing difficulty in normal eating may suffer from malnutrition and a decline in immune function, further increasing their susceptibility to *Candida albicans* infections. Prolonged usage of antibiotics, hormones, or chemotherapy drugs can disrupt the normal microbiota of the oral cavity and intestine, providing favorable conditions for *Candida albicans* growth. Therefore, it is crucial to exercise caution and take preventive measures against fungal infections when treating these patients.

However, our study also has some limitations that need to be acknowledged and addressed. First, our sample size was relatively small and uneven among different groups, which might affect the statistical power and the generalizability of our findings. Therefore, future studies with larger and more balanced sample sizes are needed to confirm and extend our findings. Second, our study was a cross-sectional study, which could not establish the causal relationship between stent placement, granulation tissue formation, and microbiota. Therefore, future studies with longitudinal or interventional designs are needed to determine the causality and the temporal sequence of these factors.

In summary, as the duration of stent placement increased, there were significant changes observed in the composition of the respiratory microbiota, which may have a close association with the development of granulation tissue formation. This study offers a comprehensive insight into the alterations in respiratory microbiota following stent placement treatment in patients with malignant central airway stenosis. It also presents novel perspectives and references for optimizing and enhancing stent placement procedures and clinical anti-infection treatments.

## MATERIALS AND METHODS

### Study design and subjects

Using a cross-sectional study method, we continuously collected patients with malignant central airway stenosis who received airway stent treatment in the Department of Respiratory Medicine of Emergency General Hospital from July 2022 to March 2023. We collected their BALF for mNGS and culture. We collected their clinical data, including the following variables: gender, age, diagnosis, stent placement time, specimen collection time, stent type, granulation tissue formation occurrence after stent placement, mNGS results, routine culture results, respiratory infection occurrence when collecting specimens, diabetes status, and immunological drug use within 1 year. The inclusion criteria were as follows: (i) tracheal stenosis confirmed by high-resolution CT of the neck and chest and electronic bronchoscopy, and the tissue pathology of the stenosis confirmed as a malignant tumor. (ii) Patients with airway stenosis who received airway stent treatment by bronchoscopy. The exclusion criteria included the following: (i) patients with non-malignant airway stenosis and those who did not receive stent placement; (ii) patients who had local tissue proliferation after stent placement but confirmed by pathology as non-granulation tissue; and (iii) patients who had no follow-up.

### Sample collection

All BALF samples were collected in the bronchoscopy room. Patients were intubated with a rigid bronchoscope (10318GL, Karl Storz, Germany) under general anesthesia. During the insertion of the rigid bronchoscope, the Olympus BF-260/BF-290 fiberoptic bronchoscope (Olympus, Japan) was kept in the rigid sheath without aspiration of oral and pharyngeal secretions, ensuring the front end of the fiberoptic bronchoscope was not disturbed by the oral and pharyngeal microbiota. After fixing the top of the bronchoscope at the target site, 100 mL of room temperature saline was injected into the bronchoscope in three to four infusions, each time injecting about 20–30 mL of saline. Patients with tracheoesophageal fistula were irrigated around the orifice of the fistula. The saline in the bronchial lumen was then aspirated with a negative pressure below 100 mmHg. Twenty milliliters of the recovered liquid was discarded for the first lavage to avoid contamination, and 5–10 mL of the remaining recovered lavage liquid was reserved in a sterile collection tube. All samples were stored at –80°C within 2 h of collection.

### Nucleic acid extraction

Samples of BALF were collected from patients according to standard procedures. DNA was extracted using the TIANamp Magnetic DNA Kit (Tiangen) according to the manufacturer’s protocols. The quantity and quality of DNA were assessed using the Qubit (Thermo Fisher Scientific) and NanoDrop (Thermo Fisher Scientific), respectively.

### Library preparation and metagenomic sequencing

DNA libraries were prepared using the KAPA Hyper Prep kit (KAPA Biosystems) according to the manufacturer’s protocols. Cluster generation, template hybridization, isothermal amplification, linearization, and blocking denaturing and hybridization of the sequencing primers were performed according to the workflow specified by the service provider. Agilent 2100 (Agilent Technologies) was used for quality control, and DNA libraries were 150 bp pair-end sequenced on Illumina Novaseq 6000 (Illumina).

### Bioinformatics analysis

We used an in-house developed bioinformatics pipeline for pathogen identification. Briefly, high-quality sequencing data were generated by removing low-quality reads, adapter contamination, and duplicated and short (length < 36 bp) reads. The human host sequence was identified by mapping to the human reference genome (hs37d5) using the bowtie2 software. Reads that could not be mapped to the human genome were retained and aligned with the microorganism genome database for pathogen identification. Our microorganism genome database contained bacteria, fungi, viruses, and parasite genomic sequences (download from https://www.ncbi.nlm.nih.gov/).

### Statistical analysis

The mNGS results were analyzed using R software (v4.0.1). Chao1, Shannon index, etc., were calculated to evaluate the alpha diversity, and the Wilcoxon rank sum test was used to compare the differences in bacterial diversity between the two groups. Bray-Curtis distance was calculated to evaluate the beta diversity, and PCoA was used to achieve visualization. Multivariate analysis of variance (PERMANOVA and ADONIS) was used to evaluate the statistical differences in beta diversity between the two groups. Kruskal-Wallis rank sum test was used to identify the differences in microbial abundance at different levels between the two groups, and the filtering criteria for microbes were relative abundance > 1% and prevalence > 40%. FDR method was used to correct all *P* values, and LEfSe was used to evaluate whether the differences in microbial relative abundance between groups were statistically significant.

## Data Availability

The metagenome sequencing data generated and analyzed during the current study is available in the SRA repositories under the number PRJNA1061381.

## SUPPLEMENTAL MATERIAL

Supplemental figures spectrum.03472-23-s0001.pdf Fig. S1 and S2.

ASM does not own the copyrights to Supplemental Material that may be linked to, or accessed through, an article. The authors have granted ASM a non-exclusive, world-wide license to publish the Supplemental Material files. Please contact the corresponding author directly for reuse.
