# Supplementary material for: Analyzing the characteristics of respiratory microbiota after the placement of an airway stent for malignant central airway obstruction
Source: Microbiol Spectr. 2024 May 15;12(6):e03472-23. doi: 10.1128/spectrum.03472-23 (PMC11237529; doi:10.1128/spectrum.03472-23)
Supplement: Supplemental figures — Fig. S1 and S2. [file spectrum.03472-23-s0001.pdf]

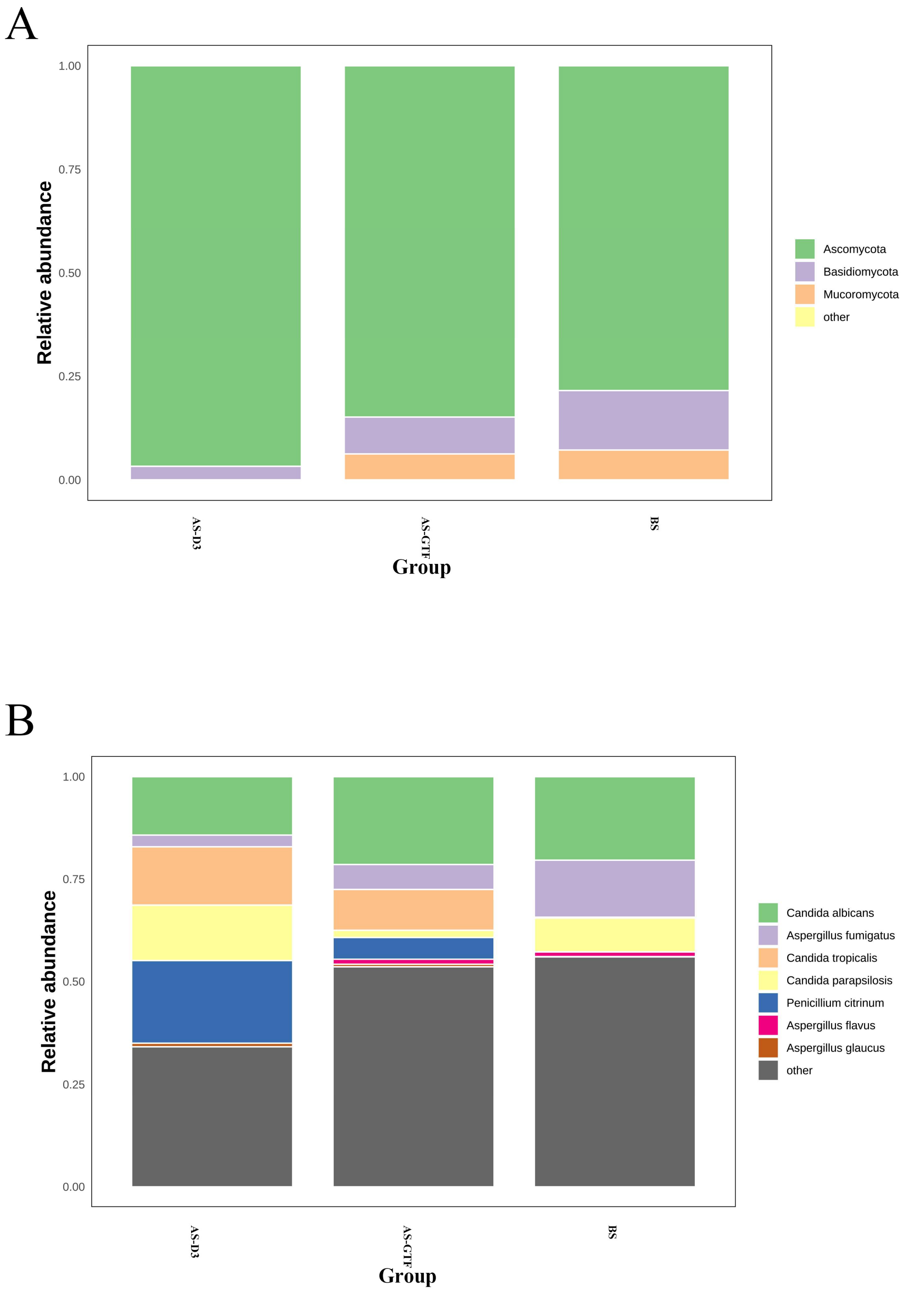

Fig. S1 Fungal microbiota composition at the phylum (A) and species (B) level in BS, AS-D3, and AS-GTF group.

A

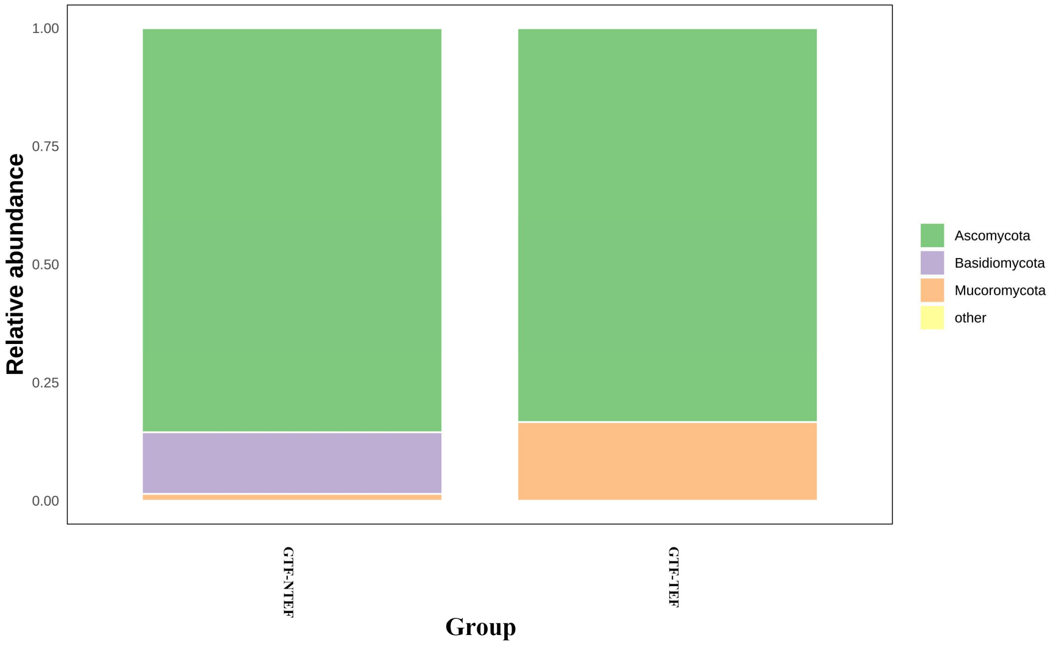
1.00
0.75
0.50
0.25
0.00
Relative abundance
GTF-NTEF
GTF-TEF
Group
Ascomycota
Basidiomycota
Mucoromycota
other

B

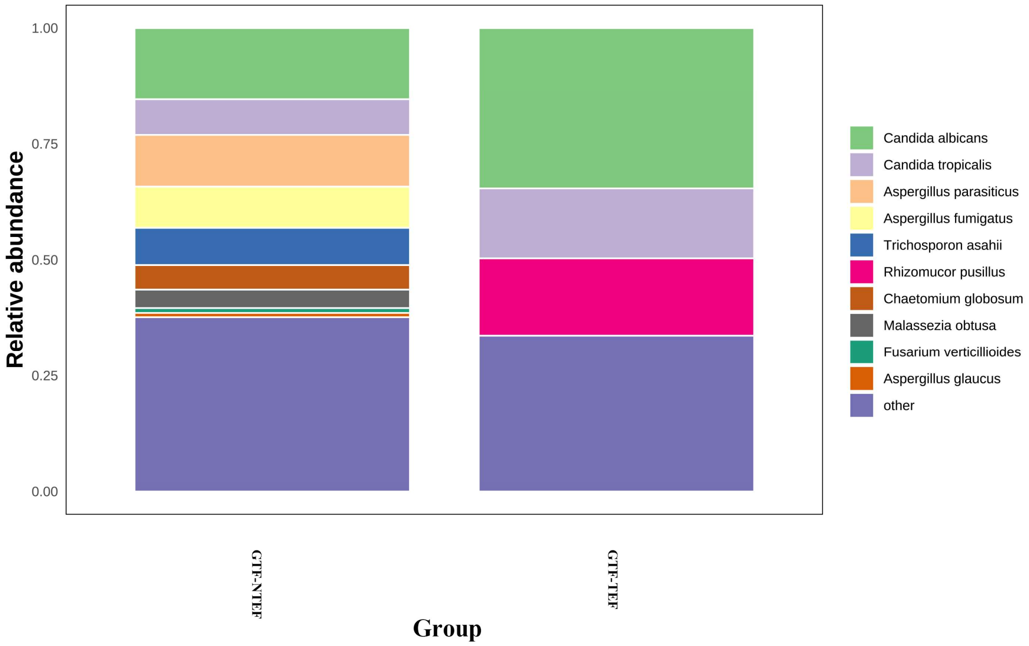
1.00
0.75
0.50
0.25
0.00
Relative abundance
GTF-NTEF
GTF-TEF
Group
Candida albicans
Candida tropicalis
Aspergillus parasiticus
Aspergillus fumigatus
Trichosporon asahii
Rhizomucor pusillus
Chaetomium globosum
Malassezia obtusa
Fusarium verticillioides
Aspergillus glaucus
other

C

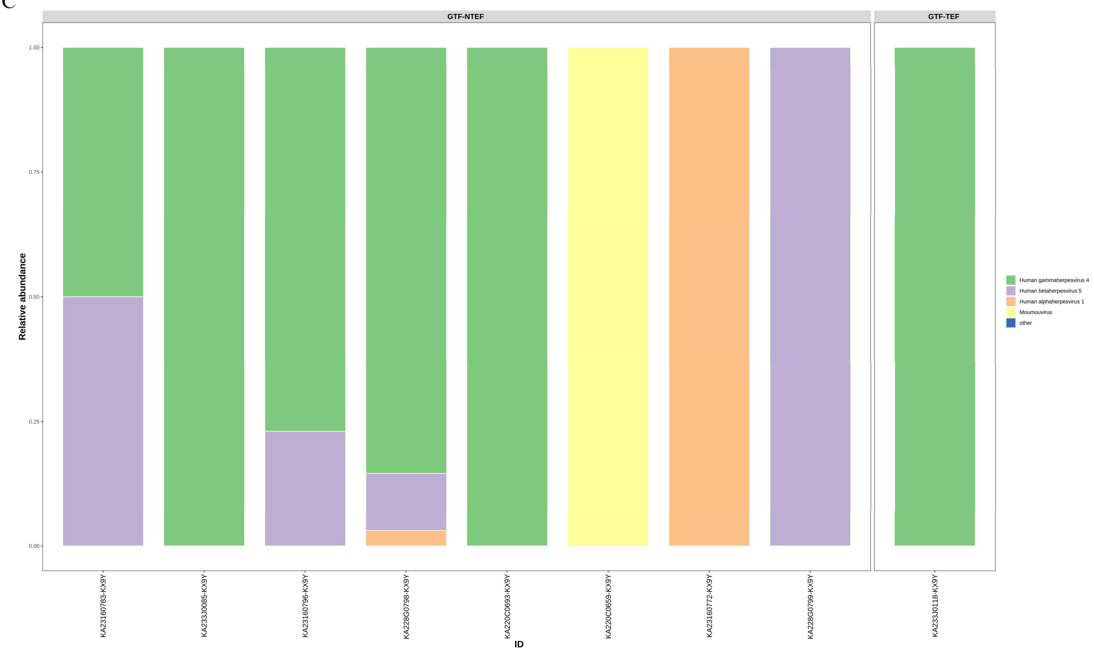

Fig. S2 Fungal microbiota composition at the phylum (A) and species (B) level and virus microbiota composition(C) at species levels in TEF and NTEF subgroups.
